# LncRNA BC200 Promotes Esophageal Squamous Cell Cancer Migration and Invasion and Can Regulate ATF4 Expression

**DOI:** 10.3389/fonc.2020.01392

**Published:** 2020-08-20

**Authors:** Ruihua Zhao, Xinguang Cao, Shuiling Jin, Rui Li, Qian Zhong, Miao Jiang, Jinming Han, Changqing Guo, Hong Zong

**Affiliations:** ^1^Department of Oncology, The First Affiliated Hospital of Zhengzhou University, Zhengzhou, China; ^2^Department of Digestive Disease, The First Affiliated Hospital of Zhengzhou University, Zhengzhou, China

**Keywords:** BC200, esophageal squamous cell cancer, ATF4, LncRNA, metastasis

## Abstract

**Background:** The main reason for esophageal squamous cell carcinoma (ESCC) treatment failure is metastasis. Little is known about the mechanisms involved in the metastasis of ESCC, and there is a lack of effective therapeutic targets. In our previous study, we found that patients with high levels of BC200 tended to have poor prognoses.

**Methods:** First, we applied qRT-PCR to detect the expression level of BC200 in normal esophageal squamous epithelial cells and ESCC cells with different degrees of differentiation ability. Then, we changed BC200 expression by transfecting constructed lentiviruses that included BC200 shRNA (LV-BC200-shRNA, KD), negative control (CON053, NC), or BC200 gene (LV-BC200, BC200) to create BC200-deficient cell models in KYSE410 and KYSE70 cells and BC200 overexpression cell models in EC9706 cells and verified the transfection effect by qRT-PCR. Then, we examined cell migration by wound healing assay, invasion by Transwell assay, and proliferation by MTT assay and examined the metastasis ability in a xenograft mouse model. Gene expression profiling was performed to screen a panel of mRNAs following inhibition of BC200 expression. We then used ingenuity pathway analysis (IPA) to analyze the functions of the changed molecules and their interactions. The results from the microarray were validated by qRT-PCR and Western blotting.

**Results:** In this study, we found that the expression of BC200 in poorly differentiated cell lines was significantly higher than that in well-differentiated cell lines. BC200 can significantly promote the migration and invasion but not the proliferation ability of ESCC cells *in vitro* and BC200 shRNA can significantly suppress tumor metastasis *in vivo*. Our genome-wide expression profile chip showed 406 differentially expressed genes, with 91 upregulated genes and 315 downregulated genes. The upstream regulator analysis showed that ATF4 was predicted to be strongly inhibited and 21 genes were consistently inhibited by this gene. Our qRT-PCR and Western blotting data also identified the reduced expression of ATF4 and some selected downstream genes, such as SNAIL2, GADD45A, and PSAT1, as a consequence of downregulating BC200 expression in ESCC.

**Conclusion:** Our data showed that BC200 promoted the metastasis of ESCC cells and could regulate the expression of ATF4 and its downstream genes.

## Introduction

Esophageal cancer is one of the most aggressive diseases and ranks seventh in incidence and sixth in tumor-related deaths worldwide. Among esophageal cancer patients, more than 70% have esophageal squamous cell carcinoma (ESCC), and most of them are from several Eastern and Southern African countries, especially China (1). Because early diagnosis is difficult, most patients are diagnosed at an advanced stage. Even after radical surgery, radiotherapy, and multiple courses of chemotherapy, most of these patients still experience recurrence and metastasis, and the 5-year survival rate is <50% (2). The most fundamental reason for treatment failure is that the mechanism of recurrence and metastasis has not been fully elucidated and lacks effective treatments. Therefore, disclosure of the metastatic mechanism of ESCC is the key to improving the survival of ESCC patients.

Long non-coding RNAs (lncRNAs) are a type of non-coding RNA whose length is more than 200 bp. Emerging evidence has shown that lncRNAs are implicated in important life processes, such as X chromosome silencing, genomic imprinting, chromatin modification, gene transcription, and nuclear transport (3). Mounting evidence has indicated that lncRNAs play an important role in the development and progression of human tumors, including ESCC. For example, the expression of lncRNA UCA1 in ESCC tissues is significantly higher than that in normal tissues. The high expression of UCA1 is associated with the poor prognosis of ESCC patients. Downregulating the expression of UCA1 can inhibit the proliferation, invasion, and migration of ESCC cells (4, 5). Besides, the high expression of another lncRNA, HOTAIR, is significantly related to the poor prognosis of ESCC patients (6). Therefore, lncRNAs may serve as critical biomarkers for tumor diagnosis and prognosis and may also serve as treatment targets.

BC200 is a non-coding RNA with a length of ~200 bp that is selectively expressed in the nervous system (7). However, a study found that BC200 expression can be detected in tumor tissues of the cervix, breast, lung, esophagus, ovary, stomach, and skin but not in adjacent normal tissues (8). Several studies have found that BC200 acts as an oncogene in some cancers, such as lung cancer and colorectal cancer, and studied the functional mechanism of BC200 (9–11). A recent report showed that BC200 can play a role in promoting the proliferation, migration, and invasion of luminal and triple-negative breast cancer cells partly by regulating CALM2 expression (12). Our previous research also showed that BC200 expression was increased significantly in ESCC tissues compared with adjacent normal tissues, and patients with a high level of BC200 had shorter disease-free survival (DFS) and overall survival (OS) than those with a low level of BC200. However, the biological functions and mechanisms of BC200 in ESCC remain unclear. In this study, we found that the expression of BC200 in poorly differentiated cell lines was significantly higher than that in well-differentiated cell lines. BC200 can significantly promote metastasis of ESCC cells. We also found that the expression of ATF4 and its downstream genes was significantly reduced as a consequence of downregulating BC200 expression.

## Materials and Methods

### Cell Cultures and Treatment

HEEC human normal esophageal epithelial cells and human ESCC cells (KYSE450, KYSE150, KYSE510, KYSE410, KYSE70, and EC9706) were obtained from the American Type Culture Collection (ATCC) and were routinely maintained in high glucose DMEM with 10% fetal bovine serum (FBS). According to the description of the Japanese Collection of Research Bioresources (JCRB) cell bank, KYSE70, KYSE150, and KYSE410 were classified as poorly differentiated cell lines, and EC9706, KYSE450, and KYSE510 were classified as well-differentiated cell lines. Cells were cultured at 37°C in a humidified incubator containing 5% CO_2_.

### Cell Transfection

The lentiviral vectors including BC200 shRNA (LV-BC200-shRNA, KD), the full length of BC200 gene (LV-BC200, BC200), or negative control (CON053, NC) were constructed using the pGCSIL-004-hU6-CMV-EGF(GV115) vector by GenePharma Co (Shanghai, China). 293T cells cultured in complete DMEM containing 10% FBS were used as lentivirus packaging cells. After cotransfection with the lentiviral vector and packaging plasmids for 48 h, we collected the media of the transfected cells and then filtered and concentrated the viral particles. The KYSE410, KYSE70, and EC9706 cells were seeded into a six-well plate at a density of 4 × 10^4^ cells per well and then cultured at 37°C to 20% confluence. Then, the cells were infected with the constructed lentiviruses (KD: 3 × 10^8^ TU/ml; NC: 4 × 10^8^ TU/ml; BC200: 3 × 10^8^ TU/ml). After infection for 12 h, the culture medium was changed to complete medium. A fluorescence microscope was used to observe the expression of green fluorescent protein (GFP) to estimate the infection efficiency after infection for 72 h. If the infection rate reached 70%, quantitative real-time reverse transcription PCR (qRT-PCR) was used to identify the expression change of BC200. The infected cells were used for functional and mechanistic experiments.

### MTT Assay

Cell proliferation was assessed by MTT assay using the MTT Cell Proliferation Assay Kit (Trevigen, Gaithersburg, MD, USA). Cells were seeded into 96-well plates at a density of 2 × 10^6^/well, and each group had five replicates. MTT was measured at 24, 48, 72, 96, and 120 h to measure the proliferation ability of the transfected cells.

### Wound Healing Assay

Infected cells were seeded in a six-well cell culture plate at a density of 3 × 10^3^/well and cultured until they reached 90% confluence. The wound was created with a 200-μl pipette tip at the central area of the cell monolayers. After that, the debris was removed, and the cells were washed with serum-free medium three times. Then, photographs were taken immediately. Cells were then continuously cultured with serum-free medium for 48 h. Photographs were collected at 24 and 48 h after wounding. The wounded areas were calculated using ImageJ software. The migration rate was calculated by the ratio of migration distance at different observation time points to the width value at 0 h.

### Transwell Assay

Transwell chambers of 24-well plates were precoated with Matrigel (50 μl of Matrigel, diluted 1:3 in serum-free medium). Infected cell suspensions were prepared in serum-free medium at a density of 2.0 × 10^5^/ml. A total of 500 μl of cell suspension was added to the upper chamber, and 750 μl of medium containing 30% FBS was added to the lower chamber. After culturing for 48 h, the cells in the upper chamber were removed, and the invading cells adhered to the lower surface of the Transwell membrane were fixed with 4% paraformaldehyde for 15 min and stained with 0.005% crystal violet for 15 min. Penetrating cells were photographed and counted under a 200× optical microscope in 10 randomly selected visual fields.

### Tumor Xenograft Assay

Female BALB/c nude mice (5 weeks) were obtained from SLAC Laboratory Animal Co (Shanghai, China). All animal experiments were carried out following the UMMC Animal Care protocol and approved by the Ethics Committee of the First Hospital of Zhengzhou University. KYSE70 cells with luciferase reporters and either BC200-shRNA (KD) or negative control (NC) were harvested and then injected via the tail vein (2 × 10^6^ cells per mouse). Each group contained six nude mice. Tumor growth and metastasis were monitored weekly after the injection using a Lumina LT small-animal live imaging system (Perkin Elmer, Waltham, MA, USA). Animals were injected intraperitoneally with 10 μg/g D-luciferin (15 mg/ml) 15 min before *in vivo* imaging and then anesthetized with an intraperitoneal injection of 0.7% pentobarbital sodium (10 μg/g per mouse). The total radiant efficiency of each mouse was recorded and analyzed. After metastasis was successfully detected, mice were euthanized by cervical dislocation, and the presence of tumors was confirmed by dissection. Lung and liver tissues were resected. After weighing, tissues were stored in liquid nitrogen for further use.

### Gene Expression Profiling

Total RNA was extracted from cells by TRIzol reagent and analyzed by an Agilent 2100 Bioanalyzer (Agilent, Thermo Fisher Scientific, USA). Then, biotin-labeled amplified RNA (aRNA) was prepared using the GeneChip 3′ IVT Express Kit according to the manufacturer's instructions (Affymetrix, Thermo Fisher Scientific, Waltham, MA, USA). After fragmentation, the labeled samples were then hybridized with the GeneChip prime view human chip probes, washed and dyed using GeneChip Hybridization Oven 645 and GeneChip Fluidics Station 645 according to instructions of the GeneChip Hybridization, Wash, and Stain Kit (Affymetrix, Thermo Fisher Scientific, Waltham, MA, USA). Fluorescence data were collected by using a GeneChip Scanner 3000 (Affymetrix, Thermo Fisher Scientific, USA) according to the manufacturer's recommendations. The background was subtracted from the raw data, and the signal value for each probe was considered to be detectable if the signal intensity > average of the negative control's intensity + 3 SDs of the negative control's intensity. Detectable signals were normalized to remove system-related variations by comparing them with the average signals of their internal control. An online integrated software ingenuity pathway analysis (IPA) (www.ingenuity.com) was used to predict upstream regulators that may cause the observed gene expression changes. The upstream regulatory factor can be any molecule that can affect gene expression. It covers all molecular types, including transcription factors, cytokines, small RNAs, receptors, kinases, chemical molecules, and drugs. IPA uses the activation *z* score algorithm to predict the activation or inhibition of upstream regulators and reduces the significant predictions due to random data. *z* score > 2 means that the regulator is significantly activated, and *z* score < −2 means that the regulator is significantly inhibited.

### Quantitative RT-PCR

Total RNA was extracted from cells by using TRIzol reagent. The RNA was subjected to RT using the Prime-Script RT Reagent Kit according to the manufacturer's instructions (Takara Biotechnology Co, Dalian, China). Quantitative real-time reverse transcription PCR (qRT-PCR) was performed using SYBR Premix Ex Taq TM (Takara Biotechnology Co, Dalian, China) in a LightCycler 480 system (Roche Diagnostics, Indianapolis, IN, USA) according to the manufacturer's instructions. All reactions were performed in a 20-μl reaction volume and run in triplicate. Primers for ATF4, SNAIL2, GADD45A, PSAT1, and GAPDH were obtained from GenePharma Co., Ltd (Shanghai, China). The primers for PCR were as follows:

ATF4: forward, 5′-TTCACCTTCTTACAACCTCTTCC-3′;

reverse, 5′-AGTCTGGCTTCCTATCTCCTTC-3′;

SNAIL2: forward, 5′-CAAGGACACATTAGAACTCACAC-3′;

reverse, 5′-CTACACAGCAGCCAGATTCC-3′;

GADD45A: forward, 5′-GAGAGCAGAAGACCGAAAGG-3′;

reverse, 5′-CAGCAGGCACAACACCAC-3′;

BC200: forward, 5′-GGATAGCTTGAGCCCAGGAGT-3′;

reverse, 5′-GGTTGTTGCTTTGAGGGAAGT-3′;

PSAT1: forward, 5′-GGGACTATAAATATCGTTCACCC-3′;

reverse, 5′- GTCATCACGGACAATCACCAC-3′;

and GAPDH: forward, 5′-TGACTTCAACAGCGACACCCA-3′;

reverse, 5′-CACCCTGTTGCTGTAGCCAAA-3′.

Following an initial denaturation at 95°C for 30 s, a total of 45 cycles of amplification were performed, with each cycle consisting of 95°C for 30 s and 60°C for 1 min. Standard curves were generated, and the relative amount of target gene mRNA was normalized to GAPDH. Data were analyzed using the 2^−ΔΔCT^ method.

### Western Blotting

Protein extracts were resolved on 8–15% SDS-PAGE gels and transferred onto PVDF membranes (Millipore, Billerica, MA, USA). The membranes were blocked with 5% nonfat dry milk in TBS containing 0.1% Tween 20 at room temperature for 1 h and incubated with antibodies against human ATF4 (1/1000, ab184909, Abcam, Cambridge, MA, USA), SNAIL2 (1/1000, #9585, Cell Signaling Technology, Danvers, MA, USA), GADD45A (1/1000, ab180768, Abcam, Cambridge, MA, USA), PSAT1 (1/1000, ab96136, Abcam, Cambridge, MA, USA), or GAPDH (1/2000, sc-32233, Santa Cruz Biotechnology, Dallas, TN, USA) overnight at 4°C, followed by incubation with the secondary peroxidase-conjugated anti-mouse (1/5000, #7076, Santa Cruz Biotechnology, Dallas, TN, USA) or rabbit IgG antibodies (1/5000, #7074, Santa Cruz Biotechnology, Dallas, TN, USA). The antigen–antibody reaction was visualized using the Pierce™ ECL Western Blotting Substrate (Thermo Fisher Scientific, Waltham, MA, USA).

### Statistics

Two-sided Student's *t*-test was used for statistical analysis, and data are presented as the mean ± SD. All *p*-values were two-tailed and considered significant at <0.05. All *in vitro* experiments were performed at least three times.

## Results

### The Expression of BC200 in Poorly Differentiated Cell Lines Was Significantly Higher Than That in Well-Differentiated Cell Lines

**Figure 1:**
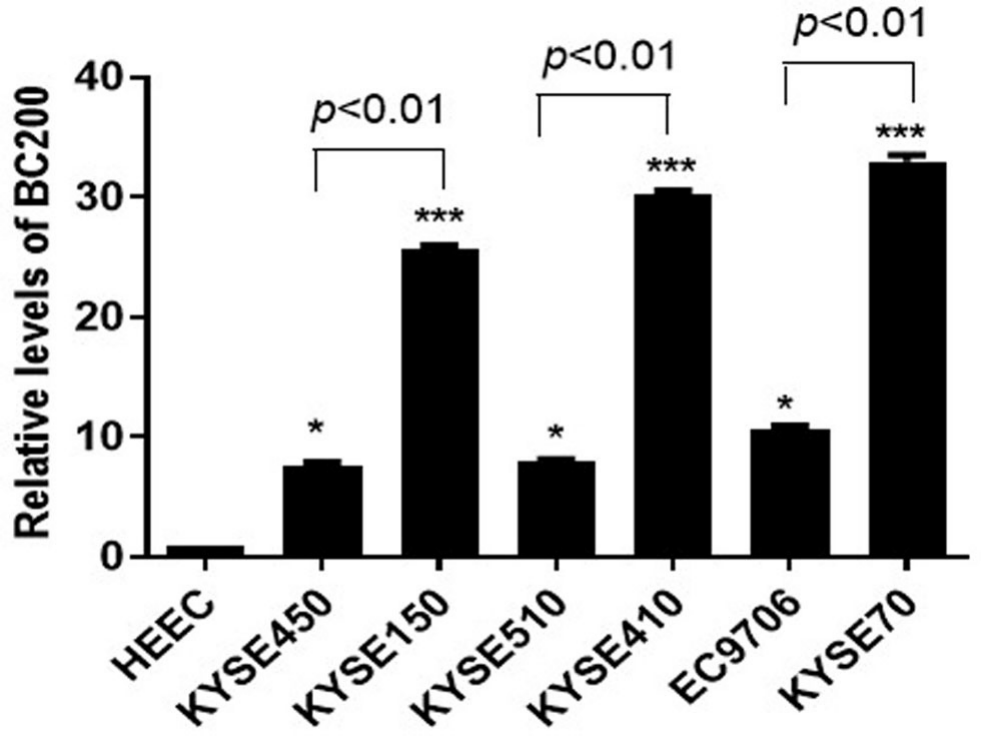
Expression of BC200 in normal esophageal squamous epithelial cells and ESCC cell lines with different degrees of differentiation and infiltration ability assayed by qRT-PCR. The relative amount of BC200 was normalized to GAPDH. Data were analyzed using the 2^−ΔΔCT^ method. The relative expression of BC200 in different cell lines was normalized to normal esophageal squamous epithelial cells. Compared with normal esophageal squamous epithelial cells, the expression level of BC200 in ESCC cell lines was significantly increased and expression levels of BC200 in the poorly differentiated cell lines KYSE70, KYSE150, and KYSE410 were significantly higher than the differentiated cell lines EC9706, KYSE450, and KYSE510. Data are expressed as representative of three independent experiments. *p < 0.05, ***p < 0.01; error bars correspond to the mean ± SD.

To further study the function of BC200 in ESCC, we applied the qRT-PCR method to detect the expression level of BC200 in normal esophageal squamous epithelial cells and ESCC cells with different degrees of differentiation and infiltration ability. Our results showed that compared with normal esophageal squamous epithelial cells, the expression level of BC200 in esophageal cancer cells was significantly increased by ~7–33 times. Further comparing the expression levels of BC200 with different differentiated cells, we found that the expression levels of BC200 in the poorly differentiated cell lines KYSE70, KYSE150, and KYSE410 were significantly higher (3–4 times) than those of the matched differentiated cell lines EC9706, KYSE450, and KYSE510 (Figure 1).

### BC200 Promoted the Migration and Invasion but Not the Proliferation Ability of ESCC Cells

**Figure 2:**
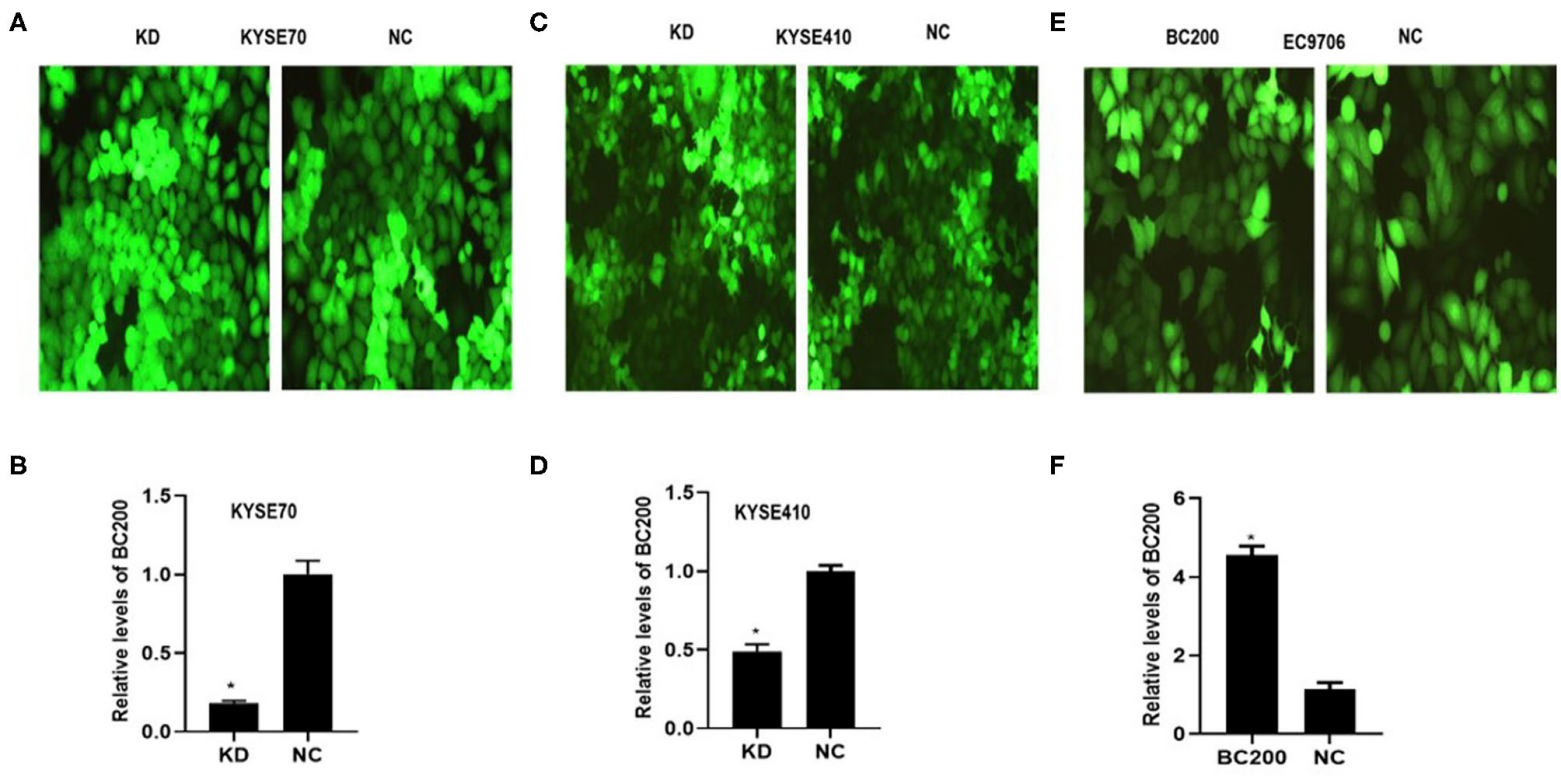
The efficiency of LV-BC200-shRNA (KD)-mediated knockdown of BC200 in ESCC KYSE70 and KYSE410 cells and LV-BC200 (BC200) mediated overexpression of BC200 in EC9706 cells. After transduction of lentiviral vectors including BC200 shRNA (LV-BC200-shRNA, KD) or negative control (CON053, NC) in KYSE70 cells and KYSE410 cells and BC200 gene (LV-BC200, BC200) in EC9706 cells, fluorescence microscope was used to observe the expression of green fluorescent protein (GFP) to estimate the infection efficiency. Fluorescence microscope pictures (200× magnification) that were taken after 72 h of transduction in KYSE70 cells **(A)**, KYSE410 cells **(C)**, and EC9706 cells **(E)**. If the infection rate reached 70%, expression of BC200 was then assessed by qRT-PCR (relative to GAPDH) in KYSE70 cells **(B)**, KYSE410 cells **(D)**, and EC9706 cells **(F)**. The data showed that BC200 expression was significantly downregulated in the KD group compared with the NC group and increased in the BC200 groups compared with the NC group. Data are expressed as representative of three independent experiments. *p < 0.05; error bars correspond to the mean ± SD.

**Figure 3:**
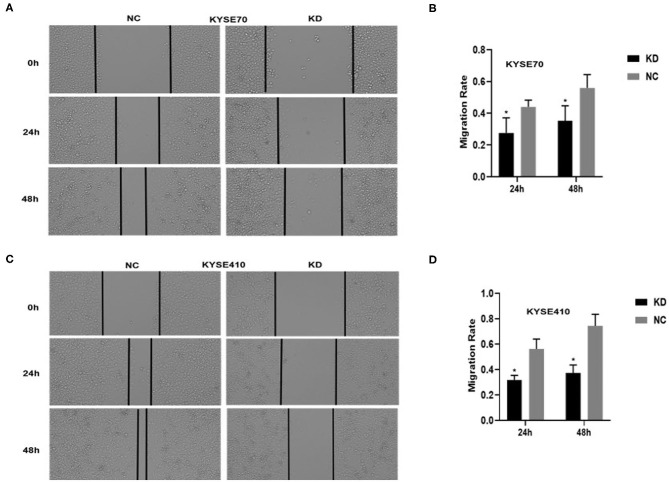
BC200 knockdown reduced the migration of ESCC cells. ESCC cells transfected with BC200 shRNA (KD) or negative control (NC) were assessed by wound healing assay. Transduced cells were cultured until they reached 90% confluence and then were wounded. Photographs were collected at 0 h, 24 h, and 48 h after wounding. **(A,C)** Representative photos of wound healing (20× magnification) in KYSE70 **(A)** and KYSE410 **(C)** cells. **(C,D)** Quantitative analyses of the migration rate in KYSE70 **(C)** and KYSE410 **(D)** cells. The migration rate was calculated by the ratio of migration distance at different observation time points to the width value at 0 h. Our data showed that the migration rate of KD cells was reduced to 30–40% of that of NC cells. Data are expressed as the mean ± SD of triplicates; *p < 0.05.

**Figure 4:**
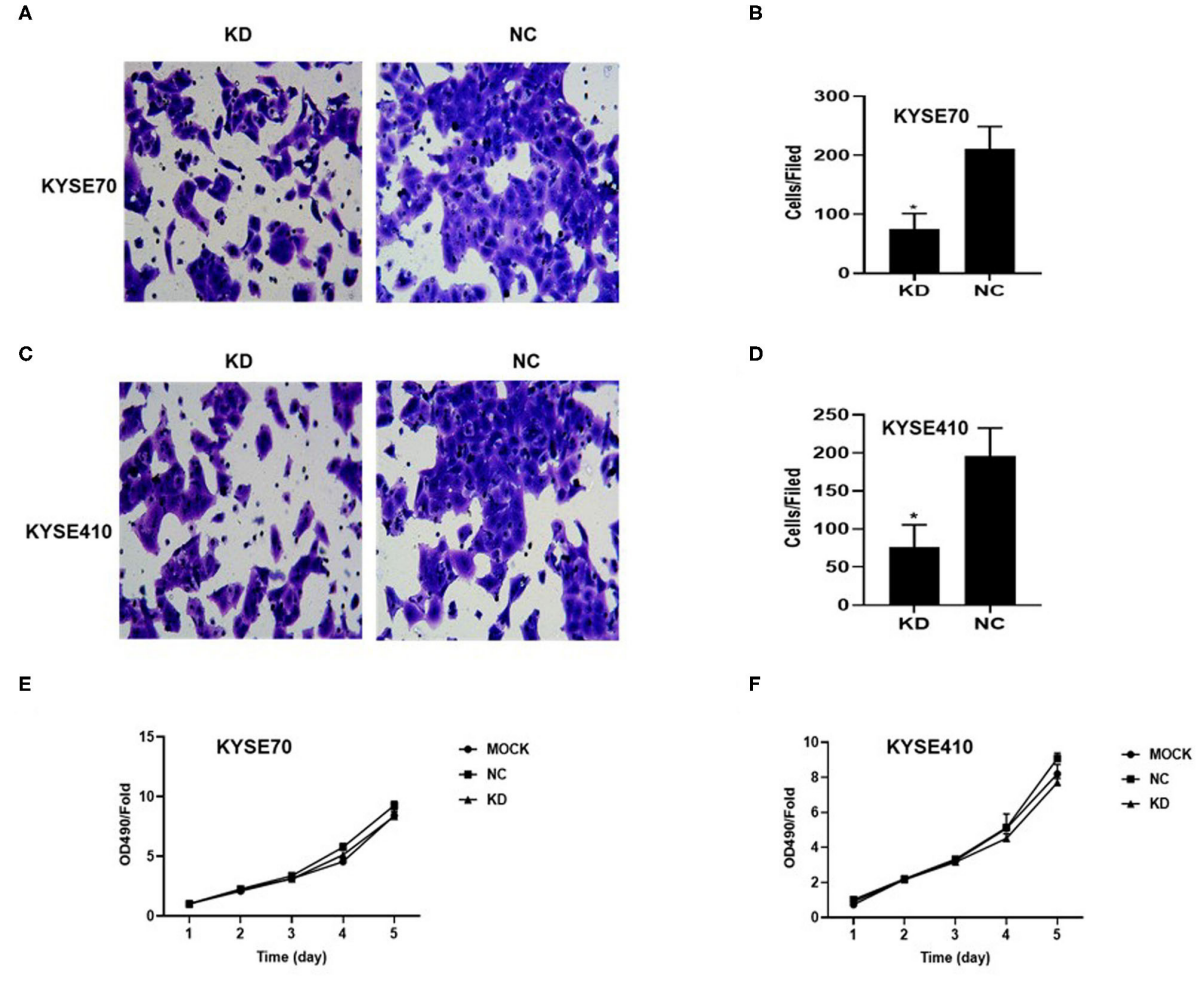
BC200 knockdown reduced the migration but not the proliferation of ESCC cells. ESCC cells transfected with BC200 shRNA (KD) or negative control (NC) were assessed by Boyden chambers coated with Matrigel **(A–D)** and MTT assay **(E,F)**. **(A,C)** Representative photos of KYSE70 **(A)** and KYSE410 **(B)** cells are shown at 200× magnification under an inverted microscope. The migrated cells were counted from 10 randomly chosen fields. The data indicated that the invasion ability of KD cells was significantly decreased than that of NC cells. *p < 0.05. Error bars correspond to the mean ± SD. **(E,F)** The proliferation of infected cells was measured at 24 h, 48 h, 72 h, 96 h, and 120 h by MTT assay. Viability was measured relative to untreated cells at each time point. It showed that the proliferation ability was not significantly different between the two groups. Data are expressed as the mean ± SD of triplicates.

**Figure 5:**
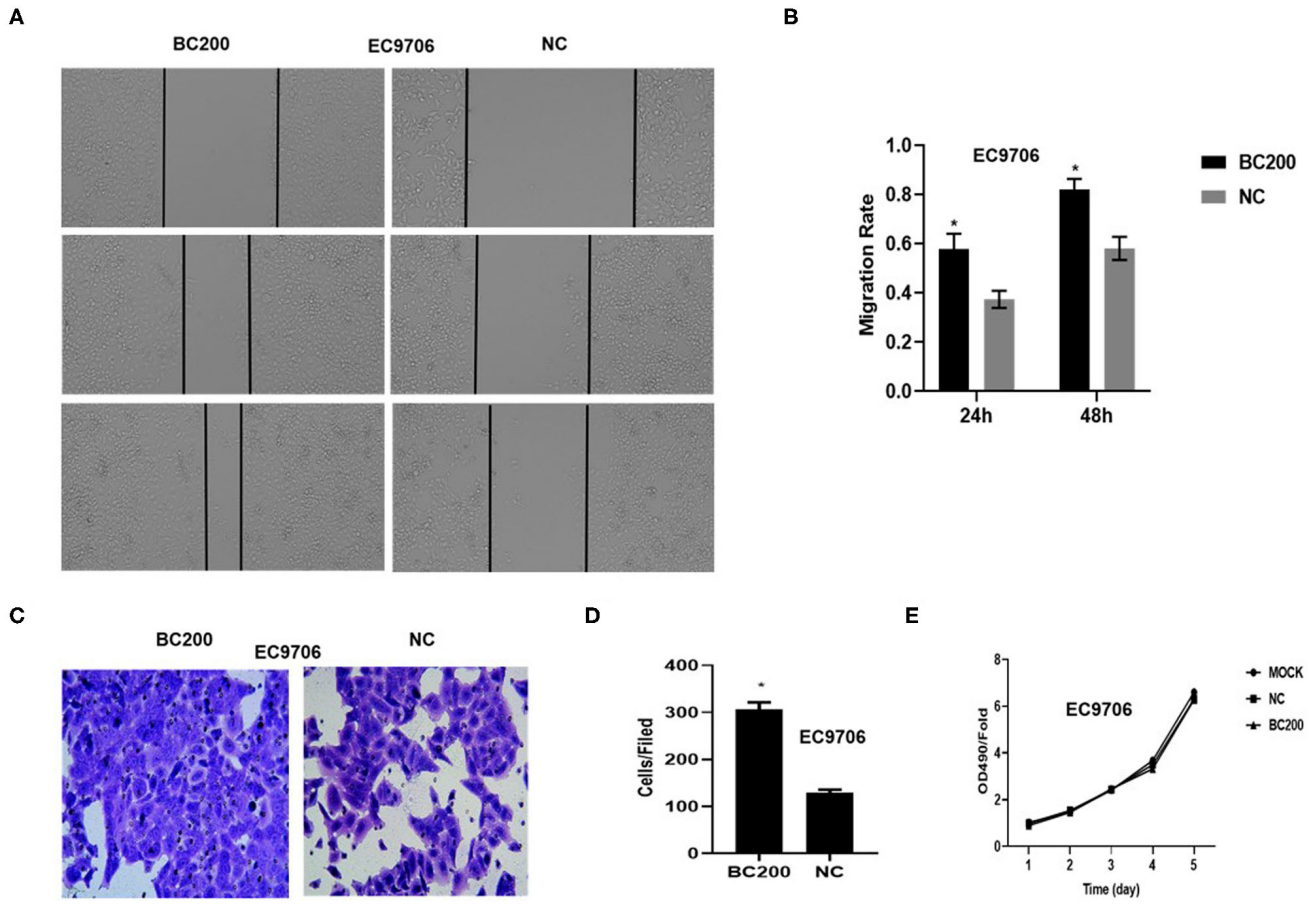
BC200 overexpression promoted the migration but not the proliferation of ESCC cells. ESCC cells EC9706 transfected with BC200 or negative control (NC) were assessed by wound healing assay **(A,B)**, Boyden chambers coated with Matrigel **(C,D)** and MTT assay **(E)**. Transduced cells were cultured until they reached 90% confluence and then were wounded. Photographs were collected at 0, 24, and 48 h after wounding. **(A)** Representative photos of wound healing (20× magnification) in EC9706 cells. **(B)** Quantitative analyses of the migration rate in EC9706 cells. The migration rate was calculated by the ratio of migration distance at different observation time points to the width value at 0 h. Our data showed that the migration rate of BC200 cells was increased to about 1.5-fold of that of NC cells. Data are expressed as the mean ± SD of triplicates; *p < 0.05. **(C)** Representative photos of EC9706 cells are shown at 200× magnification under an inverted microscope. The migrated cells were counted from 10 randomly chosen fields. The data indicated that the invasion ability of BC200 cells was significantly increased than that of NC cells. *p < 0.05. Error bars correspond to the mean ± SD. **(E)** The proliferation of infected cells was measured at 24, 48, 72, 96, and 120 h by MTT assay. Viability was measured relative to untreated cells at each time point. It showed that the proliferation ability was not significantly different between the two groups. Data are expressed as the mean ± SD of triplicates.

To determine whether BC200 contributes to ESCC cell metastasis, we created BC200-deficient cell models in KYSE410 and KYSE70 cells and BC200 expression cell models in EC9706 cells. Cells were infected with constructed lentiviruses that included BC200 shRNA (LV-BC200-shRNA, KD) or negative control (CON053, NC) or BC200 gene (LV-BC200, BC200) expressing GFP, and then the transfection efficiency was verified by qRT-PCR. A fluorescence microscope was used to observe the expression of GFP to estimate infection efficiency. If the infection rate reached 70%, qRT-PCR was used to identify the expression change of BC200. The data showed that BC200 expression was significantly downregulated in the KD group compared with the NC group, by ~81.7% and 51% in the KYSE70 and KYSE410 cell lines, respectively. The expression of BC200 was increased about four times in the BC200 group than in the NC group in the EC9706 cell lines (Figure 2). After BC200 was knocked down or overexpressed, cell invasion and migration ability were tested using wound healing and Transwell assays. Our wound healing assays showed that the migration rate of KD cells was reduced to 30–40% of that of NC cells; however, the migration rate of BC200 was increased to about 1.5-fold compared to that of NC cells (Figures 3, 5A,B). Cell invasion assays were performed using Boyden chambers coated with Matrigel. The data indicated that the invasion ability of KD cells was significantly reduced to ~60% of that of NC cells and the invasion ability of BC200 group was significantly increased to about 2-fold of that of NC cells (Figures 4A–D, 5C,D). However, our MTT cell proliferation assay over 120 h showed that the proliferation ability was not significantly different neither between KD and NC groups nor between BC200 and NC groups, suggesting that BC200 did not affect the proliferation of ESCC cells (Figures 4E,F, 5E). Therefore, our data indicated that BC200 can promote migration and invasion but not the proliferation ability of ESCC cells.

### BC200 Knockdown Suppressed Tumor Metastasis in a Xenograft Mouse Model

**Figure 6:**
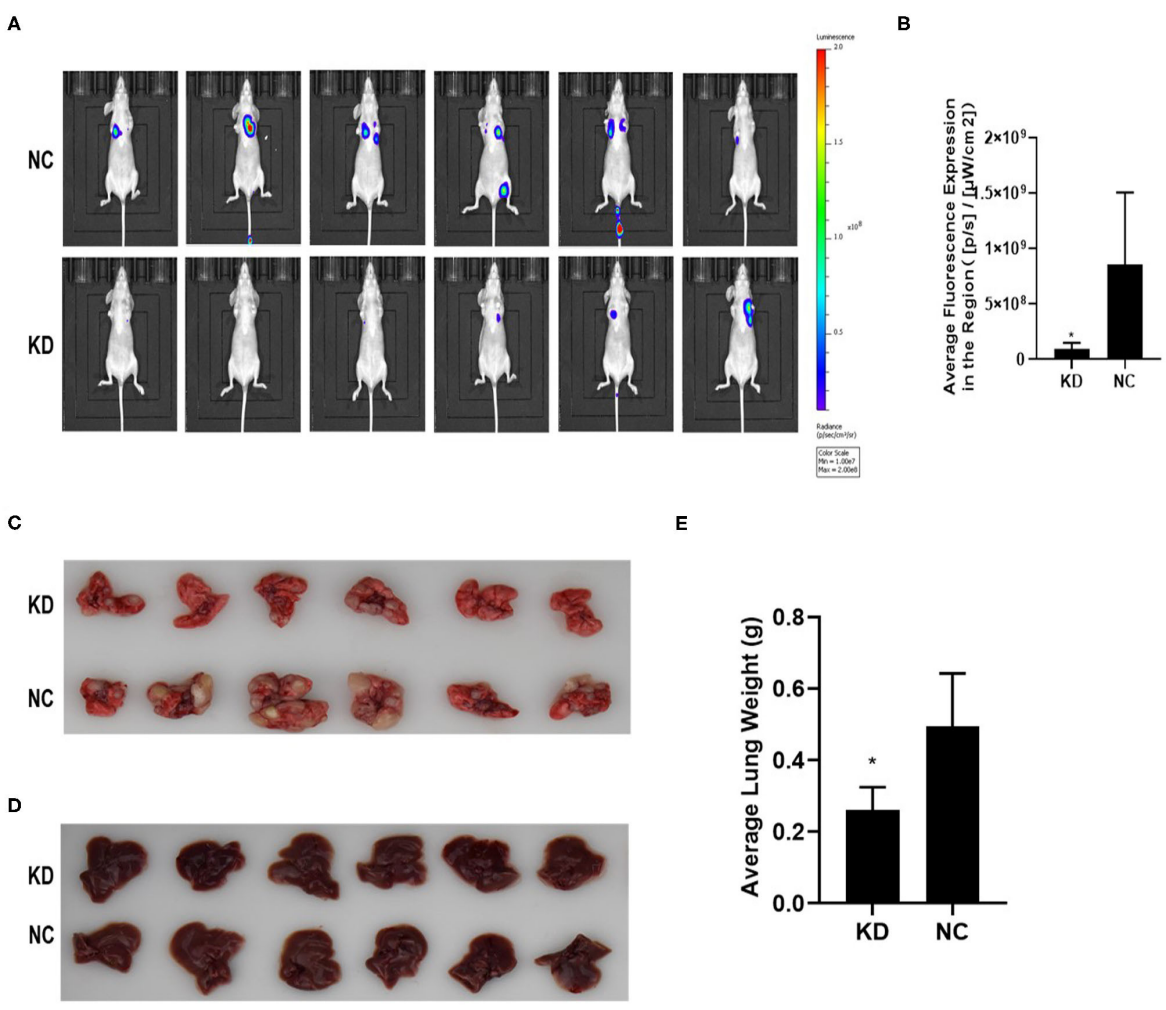
BC200 knockdown suppressed tumor metastasis *in vivo*. KYSE70 cells harboring either BC200 shRNA (KD) or negative control (NC) were injected via the tail vein. The total radiant efficiency of each mouse was recorded, and then we dissected the mice and resected the lung and liver tissues after 61 days of injection. Metastasis was detected in all the animals by the small animal live imaging system. The total fluorescence expression of the KD group was significantly reduced to 10% of that of the NC group. The metastasis rate of the lung was 100%, but no metastases in the liver. Due to a large number of different size metastases in the NC and KD groups, statistical analysis of the number and weight of metastatic lesions could not be performed. Thus, we compared the weight of the lungs between the two groups, and the data showed that compared with the NC group, the lung weight of the KD group was significantly decreased. **(A)** Photos were taken via the small-animal live imaging system. **(B)** Quantitative analyses of total radiant efficiency in different groups. Data are expressed as the mean ± SD; *p < 0.05. **(C)** Photos of the lung tissues of mice covered with metastases. **(D)** Photos of the liver tissues of mice that had no metastases. **(E)** Quantitative analyses of the lung weight between the KD and NC groups. Data are expressed as the mean ± SD; *p < 0.05.

KYSE70 cells harboring either BC200 shRNA (KD) or negative control (NC) with luciferase reporters were injected via the tail vein, and tumor metastasis was monitored weekly after the injection. The total radiant efficiency of each mouse was recorded. The mice experienced no major weight loss during the experiment. All mice seemed healthy when sacrificed. After 61 days of injection, metastasis was detected in all the animals by the small animal live imaging system. We found that the total fluorescence expression of the KD group was significantly reduced to 10% of that of the NC group (Figure 6). Furthermore, we dissected the mice and resected the lung and liver tissues to confirm metastasis. We observed no metastases in the liver. However, all the lungs were covered with metastases, with a metastasis rate of 100%. Due to a large number of different size metastases in the NC and KD groups, statistical analysis of the number and weight of metastatic lesions could not be performed. The weight of the lungs was directly proportional to the number of metastases. The number of metastases can be indirectly reflected by measuring the weight of the lungs. Then, we compared the weight of the lungs between the two groups, and the data showed that compared with the NC group, the lung weight of the KD group was significantly decreased (Figure 6). In short, our data support that inhibition of BC200 in ESCC KYSE70 cells can suppress tumor metastasis *in vivo*.

### Bc200 Knockdown Reduced the Expression of Atf4 and Its Downstream Genes

**Figure 7:**
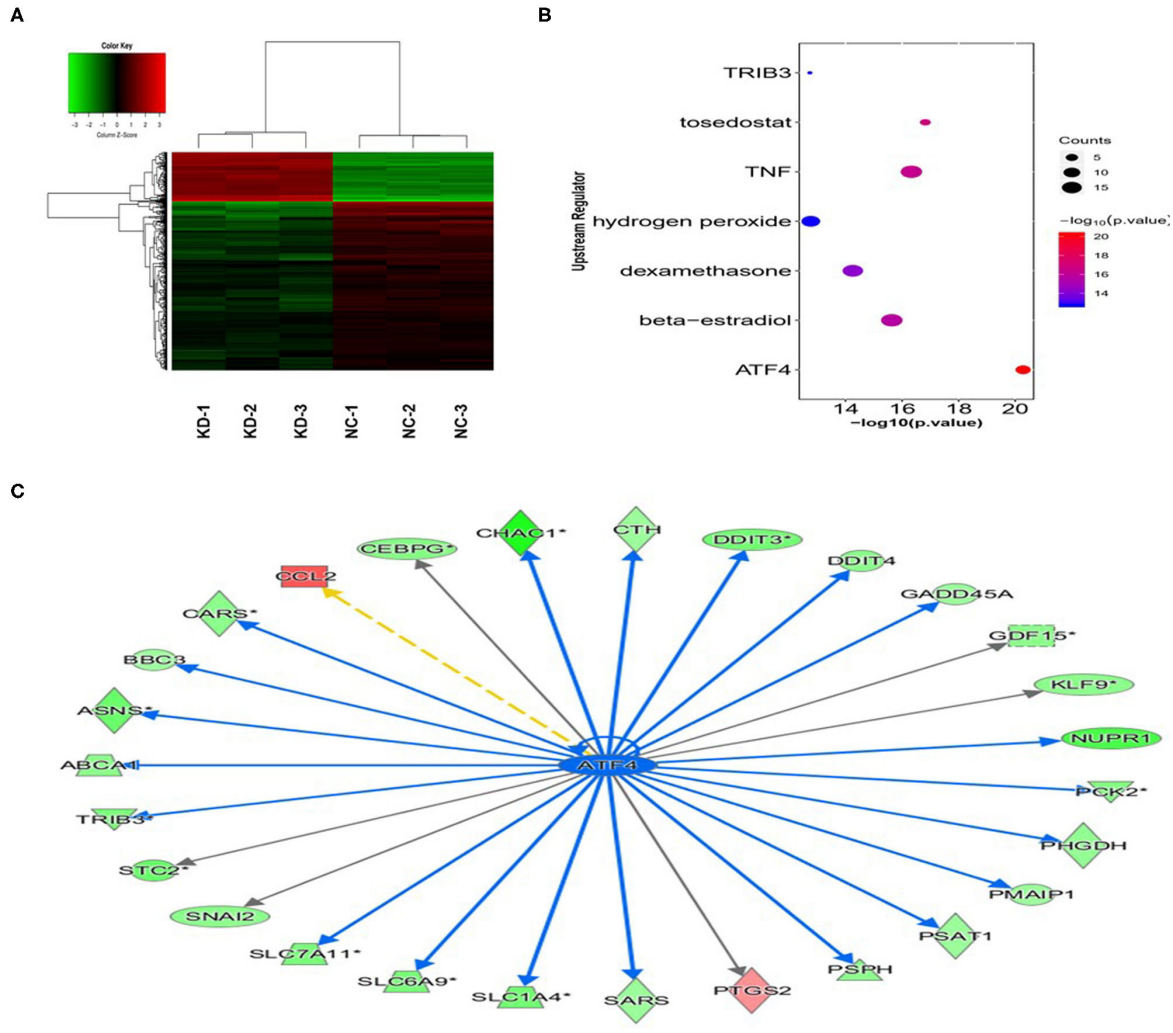
Gene expression change after BC200 knockdown. Gene expression profile analysis to screen a panel of differentially expressed genes after inhibiting the expression of BC200 in KYSE70 cells. The screening criteria for significant differential gene expression between the KD and NC groups included a fold change >1.5 and a *p*-value <0.05. **(A)** The cluster map of differentially expressed genes in all samples. Red indicates the signal value of upregulated genes, and green indicates the signal value of downregulated genes. **(B)** Bubble chart of selected upstream regulators predicted by IPA software. IPA software was used to predict the activation or inhibition of upstream regulators. According to the *z* score, −4.119, activated transcription factor 4 (ATF4) was predicted to be strongly inhibited. **(C)** The interaction between ATF4 and its directly related downstream molecules according to the IPA analysis. The orange line indicates consistent activation, the blue line indicates consistent suppression, and the yellow line indicates inconsistent expression. The gray line indicates that there is no prediction information related to the expression status in the data set.

**Figure 8:**
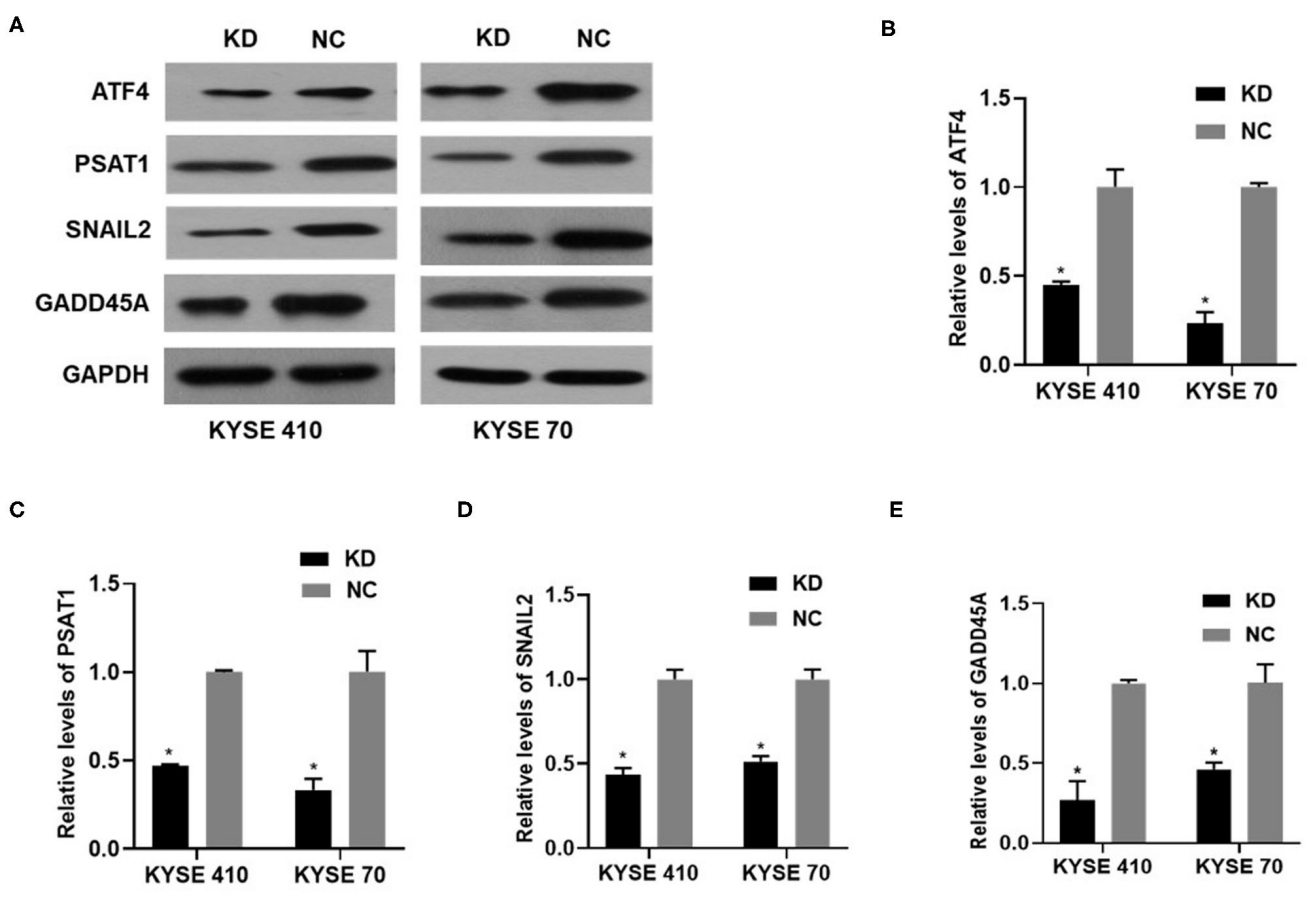
The expression of ATF4/PSAT1/SNAIL2/GADD45A protein and mRNA in KYSE410 and KYSE70 cells transfected with BC200 shRNA (KD) or negative control (NC) was measured by Western blotting **(A)** and qRT-PCR assays **(B–E)**. Data showed that the mRNA and protein expression of all these genes was significantly reduced as a consequence of downregulating BC200 expression in ESCC KYSE70 and KYSE410 cells. The relative amount of BC200 was normalized to GAPDH. Data were analyzed using the 2^−ΔΔCT^ method. The relative expression of BC200 in KD was normalized to NC cells. Data are expressed as representative of three independent experiments. *p < 0.05. Error bars correspond to the mean ± SD.

We performed gene expression profile analysis in KYSE70 cells harboring either BC200 shRNA (KD) or negative control (NC) to screen a panel of differentially expressed genes. The screening criteria for significant differential gene expression between the KD and NC groups included a fold change greater than 1.5 and a *p* value of less than 0.05. According to the above criteria, there were 406 differentially expressed genes between the KD group and the NC group, with 91 genes upregulated and 315 genes downregulated (Figure 7). Based on the gene expression data, we then performed upstream regulator analysis using IPA software to predict upstream transcriptional regulators that may cause the observed gene expression changes. IPA uses the activation *z* score algorithm to predict the activation or inhibition of upstream regulators and reduces the significant predictions due to random data. Activated transcription factor 4 (ATF4) was predicted to be strongly inhibited, fold change 1.747, *z* score = −4.119, and 21 genes were consistently inhibited by this gene (Figure 7). Then, qRT-PCR and Western blotting were used to verify the expression change of ATF4 and some selected downstream genes, such as SNAIL2, GADD45A, and PSAT1. The data showed that the mRNA and protein expression of all these genes was significantly reduced as a consequence of downregulating BC200 expression in ESCC KYSE70 and KYSE410 cells (Figure 8). Current research shows that ATF4 plays a key role in the integrated stress response of tumor cells and can promote tumor invasion and metastasis (13). Our research showed that BC200 promotes the invasion and migration of ESCC cells potentially by reducing the expression of ATF4 and its downstream genes.

## Discussion

Several studies have identified the important function and mechanism of BC200 in different kinds of cancers, suggesting that BC200 plays an important role in the progression of several cancers. Most studies have found that BC200 acts as an oncogene. For example, a study showed that the expression level of BC200 in non-small-cell lung cancer tissues is significantly higher than that in adjacent tissues (11). Further studies have found that c-Myc can upregulate BC200 in cancer tissues and cell lines by binding to the promoter region of BC200 (14). Another study confirmed that BC200 can promote the invasion and metastasis of colon cancer by upregulating the expression of salicylic acid receptor NPR3 (10). However, there are still conflicting results. A study reported that BC200 expression is significantly downregulated in ovarian cancer and that knockdown of BC200 can promote proliferation but not migration or invasion of ovarian cancer cells (15). We previously demonstrated that the expression of BC200 was increased significantly in ESCC tissues compared with adjacent normal tissues and that patients with a high level of BC200 had a shorter DFS and OS. However, the biological functions and mechanisms of BC200 in ESCC remain unclear.

Here, we expanded our previous study to investigate the role of BC200 in ESCC cells and the mechanisms of its function. In this study, we found that the expression of BC200 in poorly differentiated cell lines was significantly higher than that in well-differentiated cell lines. BC200 can significantly promote the migration and invasion but not the proliferation ability of ESCC cells *in vitro* and BC200 shRNA can significantly suppress tumor metastasis *in vivo*. To reveal the mechanism by which BC200 promotes the metastasis of ESCC, we used a genome-wide expression profile chip to detect differentially expressed genes after inhibiting BC200 expression in ESCC cells. We found 406 differentially expressed genes, of which 91 genes were upregulated and 315 genes were downregulated, and upstream regulator analysis showed that ATF4 was predicted to be strongly inhibited and 21 genes were consistently inhibited by this gene. Our qRT-PCR and Western blot data also identified the reduced expression of ATF4 and some selected downstream genes, such as SNAIL2, GADD45A, and PSAT1, as a consequence of downregulating BC200 expression in ESCC. These data triggered our interest. ATF4 is a member of the CREB/ATF family of human activating transcriptional factors and is mainly activated by translation initiation factor 2α (EIF2α). As an important transcription factor, it can be activated by various microenvironment signals, such as hypoxia, endoplasmic reticulum stress response, and the amino acid deficiency (16). Current studies have found that ATF4 is highly expressed in a variety of tumors and plays a key role in the integrated stress response of tumor cells. It can allow tumor cells to survive in the cruel microenvironment and can promote tumor invasion and metastasis (13). PSAT1 can be directly activated by ATF4 and its expression is significantly increased in a variety of tumors, including breast cancer, ESCC, etc. (17, 18). GADD45A is a stress-responsive protein that regulates genome stability, apoptosis, and immune responses, and transcription can be activated by ATF4 during many different environmental stresses (19). However, its function in ESCC cancer is still not clear. It might suggest that the effect of BC200 in ESCC is mediated through regulation of ATF4 and its downstream genes; however, that needs our further study. SNAIL2 plays an important role in inducing epithelial–mesenchymal transition (EMT) and then promoting the metastasis of tumors (20). Some of the genes that play important roles in cell metastasis and EMT, such as N-cadherin and matrix metalloproteinases (MMP2 and MMP9), were also significantly inhibited, while the expression of E-cadherin was significantly increased (data not shown). Thus, BC200 may play an important role in the EMT of ESCC cells and needs further investigation.

In summary, our study shows that the expression of BC200 in poorly differentiated cell lines is significantly higher than that in well-differentiated cell lines. Inhibiting BC200 expression in ESCC cells can significantly reduce the metastasis of ESCC cells *in vitro* and *in vivo*. We also found that the expression of ATF4 and its downstream genes was significantly reduced as a consequence of downregulating BC200 expression. This is the first study to reveal the relationship between BC200 and ATF4 in ESCC cells. Further investigation should investigate the mechanism by which BC200 regulates ATF4 and investigate whether BC200 can promote the invasion and migration of ESCC through ATF4 and its downstream pathway.

## Data Availability Statement

All datasets generated for this study are included in the article/supplementary material.

## Ethics Statement

The animal study was reviewed and approved by Ethics Committee of the First Hospital of Zhengzhou University.

## Author Contributions

HZ and CG conceived and designed the study. RZ and XC wrote the manuscript. RL performed the in vitro experiment. SJ and QZ performed the in vivo experiment. MJ revised the manuscript. JH performed the statistical analysis. All authors have read and approved the contents of the final manuscript.

## Conflict of Interest

The authors declare that the research was conducted in the absence of any commercial or financial relationships that could be construed as a potential conflict of interest.
